# Trial protocol of an open label pilot study of lisdexamfetamine for the treatment of acute methamphetamine withdrawal

**DOI:** 10.1371/journal.pone.0275371

**Published:** 2022-10-03

**Authors:** Liam S. Acheson, Nadine Ezard, Nicholas Lintzeris, Adrian Dunlop, Jonathan Brett, Craig Rodgers, Anthony Gill, Michael Christmass, Rebecca McKetin, Michael Farrell, Steve Shoptaw, Krista J. Siefried

**Affiliations:** 1 The National Drug and Alcohol Research Centre (NDARC), the University of New South Wales, Sydney, Australia; 2 Alcohol and Drug Service, St Vincent’s Hospital Sydney, Sydney, Australia; 3 The National Centre for Clinical Research on Emerging Drugs (NCCRED), c/o the University of New South Wales, Sydney, Australia; 4 New South Wales Drug and Alcohol Clinical Research and Improvement Network (DACRIN), Sydney, Australia; 5 The Langton Centre, South East Sydney Local Health District, Sydney, Australia; 6 Discipline of Addiction Medicine, the University of Sydney, Sydney, Australia; 7 Drug and Alcohol Clinical Services, Hunter New England Local Health District, Newcastle, Australia; 8 School of Medicine and Public Health, the University of Newcastle, Newcastle, Australia; 9 Clinical Pharmacology and Toxicology, St Vincent’s Hospital Sydney, Sydney, Australia; 10 St. Vincent’s Clinical School, the University of New South Wales, Sydney, Australia; 11 Next Step Drug and Alcohol Services, Perth, Australia; 12 National Drug Research Institute, Curtin University, Perth, Australia; 13 Department of Family Medicine, The University of California Los Angeles, Los Angeles, California, United States of America; PLoS ONE, UNITED STATES

## Abstract

**Introduction:**

Methamphetamine (MA) use disorder is an important public health concern. MA withdrawal is often the first step in ceasing or reducing use. There are no evidence-based withdrawal treatments, and no medication is approved for the treatment of MA withdrawal. Lisdexamfetamine (LDX) dimesilate, used in the treatment of attention deficit hyperactivity disorder and binge eating disorder has the potential as an agonist therapy to ameliorate withdrawal symptoms, and improve outcomes for patients.

**Methods:**

A single arm, open-label pilot study to test the safety and feasibility of LDX for the treatment of MA withdrawal. Participants will be inpatients in a drug and alcohol withdrawal unit, and will receive a tapering dose of LDX over five days: 250mg LDX on Day 1, reducing by 50mg per day to 50mg on Day 5. Optional inpatient Days 6 and 7 will allow for participants to transition to ongoing treatment. Participants will be followed-up on Days 14, 21 and 28. All participants will also receive standard inpatient withdrawal care. The primary outcomes are safety (measured by adverse events, changes in vital signs, changes in suicidality and psychosis) and feasibility (the time taken to enrol the sample, proportion of screen / pre-screen failures). Secondary outcomes are acceptability (treatment satisfaction questionnaire, medication adherence, concomitant medications, qualitative interviews), retention to protocol (proportion retained to primary and secondary endpoints), changes in withdrawal symptoms (Amphetamine Withdrawal Questionnaire) and craving for MA (visual analogue scale), and sleep outcomes (continuous actigraphy and daily sleep diary).

**Discussion:**

This is the first study to assess lisdexamfetamine for the treatment of acute MA withdrawal. If safe and feasible results will go to informing the development of multi-centre randomised controlled trials to determine the efficacy of the intervention.

## Introduction

An estimated 29 million people, or 0.7% of the world’s population aged between 15–64 years old, consumed amphetamine or methamphetamine (MA) in 2017 (hereafter referred to collectively as MA to reflect the most recent data on the context of the Australian market [1, 2]). The use of MA has been associated with depression and anxiety, psychosis, suicidal behaviour, cardiovascular, respiratory and dermatological conditions, blood borne virus transmission, non-fatal injury as well as overdose and death [3–6]. The global, age-standardised rate of MA/amphetamine dependence is estimated to be 96 people per 100,000 [7].

Cessation of MA use following frequent and prolonged use can result in a characteristic withdrawal syndrome, which emerges within hours to days after ceasing or reducing use [8]. This acute withdrawal syndrome is reported to last around 7–10 days and has been characterised by dysphoria, fatigue, sleep disturbances, vivid dreams, increased appetite, and psychomotor agitation or retardation, with symptoms peaking within the first 7 days [8–11]. Successful withdrawal can facilitate engagement with, and a transition to, ongoing treatment (e.g. residential rehabilitation, counselling), resulting in improved health and welfare [12, 13], and early abstinence of at least two weeks is a predictor of longer-term treatment success [14].

There are no evidence-based pharmacotherapies for the treatment of MA withdrawal [10]. Standard care generally involves management of MA withdrawal symptoms (e.g., benzodiazepines to manage anxiety and agitation) despite limited evidence of effectiveness in improving withdrawal outcomes [15]. Neurobiological models of MA withdrawal suggest that acute withdrawal symptoms (e.g. intense cravings, irritability and poor concentration) may result from dopaminergic dysfunction related to chronic exposure to high dose amphetamines [16, 17]. These changes have been correlated with mood and behavioural disturbances seen in people with chronic MA use [18]. At least partial recovery in dopamine transporter function occurs over time, however, this can take months [19]. In contrast, rodent models show the neurobiological changes that occur in withdrawal are potentially immediately reversible with administration of a stimulant [20]. Agonist therapies have been effective in the treatment of withdrawal for other substances (for example buprenorphine for opioid withdrawal [21]), and agonist-like therapies for MA dependence have shown promise in the past, particularly in their effects on craving [22].

A potential candidate drug to treat MA withdrawal is lisdexamfetamine. Lisdexamfetamine (LDX) is a pharmacologically inactive pro-drug of dexamphetamine, a central nervous stimulant which increases extracellular dopamine [23]. LDX has regulatory approval in Australia for the indications for Attention-Deficit / Hyperactivity Disorder (ADHD) and Binge Eating Disorder at doses up to 70 mg per day [24]. LDX is absorbed after oral administration and hydrolysed to inactive metabolites and active dexamphetamine by red blood cells [25]. This results in a slower onset of action/effect and lower peak dopamine concentration compared with immediate release dexamphetamine [26, 27], enabling once daily dosing.

LDX doses of up to 250mg per day have been found to be safe and tolerable in both stimulant-naïve people and a pilot study of people who use MA [23, 28, 29]. A tapering dose regimen is proposed in this protocol to allow for maximum potential attenuation of early withdrawal by providing reducing stimulant exposure through the first five days, when withdrawal symptoms are at their most severe and ensure no residual withdrawal from LDX upon ceasing treatment due to the tapering dose. As the ineffective treatment of MA withdrawal symptoms may lead to higher rates of relapse to use [30, 31], the ability for LDX as a prodrug of dexamphetamine to ameliorate withdrawal symptoms and craving for MA has the potential to improve treatment outcomes during acute withdrawal [32].

This report is a protocol for an open-label, single-arm pilot clinical trial. The protocol was developed in conjunction with SPIRIT guidelines and adheres to the Australian National Statement on Ethical Conduct in Human Research, and Good Clinical Practice guidelines [33–35].

### Objectives

This study aims to assess a five-day tapering-dose regimen of LDX for the management of acute MA withdrawal in an inpatient withdrawal setting. We hypothesise that a tapering dose of LDX will be safe and feasible to deliver during inpatient withdrawal from MA.

The primary objective is to determine the safety and feasibility of a 250mg tapering dose of LDX over five days of inpatient treatment. Secondary objectives are: (i) to describe the acceptability of the treatment regimen to participants; (ii) to describe participant retention to the protocol; (iii) to describe changes in withdrawal severity, and (iv) MA craving during and after the treatment regimen; and (v) to describe the sleep wake cycle of people with acute MA withdrawal receiving a five-day tapering dose of LDX.

## Materials and methods

### Trial design

This study is an open-label, single-arm, pilot clinical trial of a five-day tapering dose regimen of LDX for acute MA withdrawal. Participants are admitted to an inpatient withdrawal unit for up to seven days (five days of medication and two additional optional days for transition to ongoing treatment [e.g. residential rehabilitation, counselling]) and are followed up via telephone or in person once a week until Day 28 (three weeks post discharge).

### Sample size

As this is a pilot study, it is not powered to detect differences in treatment efficacy. This study will therefore seek to enrol 15 participants, conventional for studies of this nature [36, 37]. Findings will inform planning for a large-scale RCT of LDX for treatment of MA withdrawal.

### Study setting

This study is conducted at a single site inpatient alcohol and other drug withdrawal unit in Sydney, New South Wales, Australia.

### Eligibility criteria

Inclusion criteria are adults over the age of 18 years who: are presenting to inpatient drug treatment services seeking treatment for MA withdrawal; have methamphetamine use disorder (MAUD) as determined by an Addiction Medicine specialist according to the Diagnostic and Statistical Manual of Mental Disorders, 5th Edition (DSM-5) Criteria [11]; report last use of MA within 72 hours of the planned first study drug dose; have a positive urine drug screen for MA on admission, and; are willing and able to provide written informed consent and comply with the study protocol.

Exclusion criteria are: participants who are lactating, pregnant or of childbearing potential and not willing to avoid becoming pregnant during the study; expected concurrent withdrawal from alcohol, opioids, benzodiazepines, gamma-hydroxybutyrate or other gabapentinoids; known contraindications to LDX (as per the product label [24]); a medical or psychiatric comorbidity which in the opinion of a study medical officer renders a patient unsuitable for the study; or, involuntary patients. Full criteria are described in Table 1.

**Table 1:** Eligibility criteria.

Inclusion Criteria	Exclusion Criteria
Adult over the age of 18 years Presenting to inpatient drug treatment services seeking treatment for acute MA withdrawal (e.g. self-presenting to the centralised intake line, presented to the Emergency Department, presented to the Alcohol and Drug Service etc.) Methamphetamine Use Disorder as determined by an addiction medicine specialist according to the Diagnostic and Statistical Manual of Mental Disorders, 5^th^ Edition (DSM-5) criteria Last MA use within 72 hours of planned first dose of investigational product Positive urine drug screen for methamphetamines Willing and able to provide written informed consent and willing to participate in and comply with the study protocol	Women who are: lactating; pregnant; or are of childbearing potential and not willing to avoid becoming pregnant during the study Expected concurrent withdrawal from alcohol, opioids, benzodiazepines, gamma-hydroxybutyrate or other gabapentinoids Known contradiction(s) to lisdexamfetamine (as per product information [24]) including: Advanced arteriosclerosis Symptomatic cardiovascular disease including cardiac arrhythmia, ischaemic heart disease Moderate to severe hypertension Hyperthyroidism Known hypersensitivity or idiosyncratic reaction to sympathomimetic amines or any of the excipients Glaucoma Agitated states such as severe anxiety, tension and agitation During or within 14 days following the administration of monoamine oxidase inhibitors (hypertensive crises may result) Pheochromocytoma Tics, Tourette’s syndrome Patients who currently exhibit severe depression, anorexia nervosa, psychotic symptoms or suicidal tendency Significant medical or psychiatric condition which, in the opinion of a study medical officer, renders a patient unsuitable for the study Involuntary patient status

**Fig 1:**
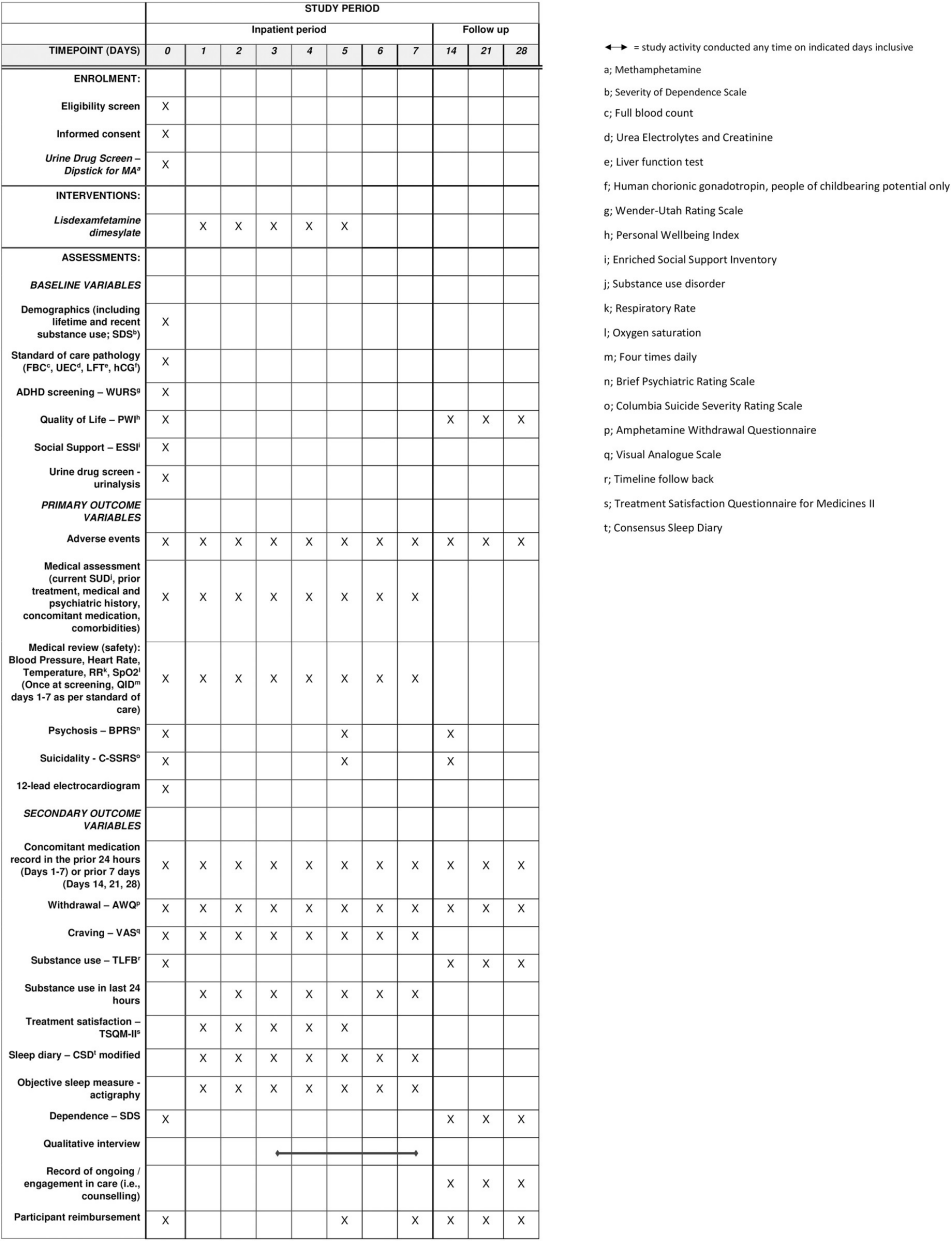
Schedule of enrolment, interventions, and assessments.

Potential participants are pre-screened by telephone or in person by a researcher prior to admission. This process facilitates orientation to the study, gauges interest, and assesses potential eligibility to participate. Individuals considered potentially eligible are invited to a formal eligibility assessment by a specialist in Addiction Medicine. The assessment schedule, including eligibility assessments, is outlined in Fig 1.

### Intervention

Participants receive a tapering dose of oral lisdexamfetamine dimesilate in 50mg capsules, beginning with 250 mg once daily (OD) on Day 1, and reduced by 50 mg per day, to 50 mg OD on Day 5 (Table 2).

**Table 2:** Study drug dosing schedule.

Study day	Day 1	Day 2	Day 3	Day 4	Day 5	Day 6	Day 7
Lisdexamfetamine	250 mg OD^a^	200 mg OD	150 mg OD	100 mg OD	50 mg OD	-	-

^a^ OD, daily; dosing should be within +/- 1 hour of scheduled dosing time

LDX at a dose of 250 mg is equivalent to approximately 74 mg of dexamphetamine [38], and similar doses of sustained release dexamphetamine (60-110mg) have previously been demonstrated to decrease MA withdrawal severity and cravings, and increase retention in care [39, 40].

### Data collection

#### Outcomes

*Primary outcomes*. Safety is assessed by Adverse Events (AEs) and vital statistics recorded during each study visit. AEs will be analysed and reported for seriousness, severity, causality, relatedness, and expectedness [41]. Vital signs (heart rate, blood pressure, temperature, oxygen saturation and respiratory rate) are recorded four times daily during the inpatient period. Safety is further assessed through differences in symptoms of psychosis and hostility obtained using the psychosis and hostility items of the Brief Psychiatric Rating Scale (BPRS) [42, 43], and suicidality measured by the Columbia Suicide Severity Rating Scale (C-SSRS) at Days 1 and 5 [44]. Feasibility is measured through the proportion of screen and pre-screen failures and time taken to enrol sample.

*Secondary outcomes*. Medication acceptability is measured by the Treatment Satisfaction Questionnaire for Medications II (TSQM-II) [45], medication adherence (proportion of prescribed doses taken and reasons for missing doses), proportion and type of adjuvant symptomatic medication required, and qualitative interviews. Retention is assessed by the proportion of participants retained in the study until Days 5, 7, 14, 21 and 28. Changes in subjective withdrawal severity are measured at each time point using the Amphetamine Withdrawal Questionnaire (AWQ) [46] through to Day 28. Changes in craving for MA during the inpatient period are assessed using a 100mm Visual Analogue Scale (VAS) [47]. Participant sleep is objectively monitored through continuous actigraphy (Philips Actiwatch 2^®^) and subjective sleep quality is measured with a modified Consensus Sleep Diary (CSD) [48].

To describe the sample additional baseline assessments include: the Wender-Utah rating scale (WURS), to screen for co-existing ADHD [49]; and severity of dependence assessed with the Severity of Dependence Scale (SDS) [50]. Quality of life and level of social support is measured using the Personal Wellbeing Index (PWI) and ENRICHD Social Support Inventory (ESSI), respectively [51, 52].

### Procedure

#### Day 0 (screening and eligibility)

When a potential participant is admitted to the inpatient withdrawal management unit, a researcher provides an overview of the study, reviews the participant information sheet with the patient, and answers questions as needed. Patients who are interested in participating in the study are asked to provide written informed consent. The day of admission is defined as Day 0 of the study, and after informed consent is provided participants are screened for eligibility by an addiction medicine specialist signed on to the study as an investigator. If eligible, participants are formally enrolled in the trial and a medical screening and assessment is undertaken. Baseline data including confirmation of recent MA use through a urine drug screen is collected. Usual inpatient withdrawal care (including symptomatic management and psychosocial support) is provided. Concomitant psychosocial therapy and AEs are recorded. Protocolised adjuvant symptomatic treatment (diazepam for anxiety (maximum 10mg four times daily [QID]) and olanzapine for psychosis (maximum 5mg three times daily [TDS])), as per standard of care are available alongside the experimental treatment as per standard of care in accordance with the protocol.

#### Days 1–5 (inpatient period, active medication)

Day 1 is defined as the first morning of admission, to allow for consistent morning dosing for LDX. Participants are assessed daily for safety, AEs, concomitant medication, and concomitant psychosocial therapies. Daily measures of treatment satisfaction, withdrawal severity, cravings for MA, and quality and duration of sleep are assessed.

Participants are invited to participate in a qualitative interview to examine their experiences of participating in the clinical trial to ensure any subsequent trials will include participant perspectives. Interviews are one-on-one, semi-structured, and approximately 30 minutes in duration. They are conducted by study staff trained in qualitative interviewing. One interview will take place between Days 3 and 7. Interviews are conducted in a private room, and electronically recorded and transcribed.

#### Days 6–7 (inpatient period, no medication)

Days 6–7 are optional for ongoing inpatient care and monitoring. Adjuvant medication, wraparound withdrawal services and discharge planning continue to be offered as usual. Participants will be assessed daily for AEs, concomitant medications, and concomitant psychosocial therapies.

Admission may extend beyond Day 7 if clinically indicated, independent of the study.

LDX is not available after Day 5. Ongoing standard treatment is individualised in collaboration with external treating clinicians (i.e., outpatient stimulant treatment programs, residential rehabilitation, etc.). Treatment access and engagement following the inpatient withdrawal period is recorded through to Day 28.

#### Days 14, 21 and 28 (follow-up)

Participants receive telephone and short message service (SMS) follow-up from a researcher on Days 14, 21 and 28. Safety, concomitant medication and psychosocial therapy, questionnaire completion and ongoing care will be recorded.

All AEs will be followed-up to resolution or until Day 28, whichever is the shorter, regardless of relatedness to study drug.

Participants are reimbursed with supermarket vouchers in line with standard practice [53], at Baseline ($20) and Days 5 ($30), 7($30) 14 ($30), 21 ($30) and 28 ($30) [53]. Total possible remuneration is $170.

#### Participant study discontinuation

Participants who wish to discontinue protocol treatment are offered the opportunity to continue to participate in all remaining research assessments. For those participants who revoke their consent to participate in the study, no further data are collected.

#### Study withdrawal criteria

Consistent with the safety protocols that are standard care at the enrolling site, review by an Addiction Medicine specialist (signed onto the study protocol) will occur where a participant records a single episode of systolic blood pressure >180mmHg or <100mmHg, a resting heart rate >120 beats per minute (bpm) or <50 bpm, or requires >40mg of diazepam over a 24-hour period to manage symptoms. These events are considered AEs. Any AE that is determined to be potentially treatment related and considered a risk to participant safety by a study doctor will result in withdrawal of the participant from the trial for safety concerns.

### Statistical methods

The adverse event verbatim descriptions will be classified into standardized medical terminology using the Medical Dictionary for Regulatory Activities (MedDRA) version 19.1. AEs will be coded to primary System Organ Class (SOC) and preferred term (PT) using MedDRA. Descriptive statistics will be used to analyse all AEs by causality and relatedness. Changes in vital signs will be described using medians and interquartile ranges. The proportion of screen failures and participants who commenced study drug; proportion who achieve each dose; and, proportion who are retained in the study or revoke consent will be analysed. Secondary outcomes measure: changes in medication tolerability; proportion of participants who reached primary and secondary end points; MA withdrawal and craving severity; and, quality and duration of sleep. These will be tested for statistical significance using Wilcoxon rank-sum test for non-parametric data. All tests of significance will be conducted at a two-sided alpha level of 0.05. As this is an exploratory study we will: (i) conduct analyses of observed data only; (ii) conduct sensitivity analyses which account for missing data by imputation; and, (iii) report missing data and limitations.

### Data statement

Study data are collected and managed using Research Electronic Data Capture (REDCap) [54, 55]. The REDCap database is hosted at St. Vincent’s Hospital, Sydney. REDCap is a secure, web-based application designed to support data capture for research studies. All data will be stored on a secure server for 15 years in a de-identified format and only researchers directly involved in the collection or analysis of data will have access to the data. At trial completion the de-identified database will be made available to researchers by reasonable request only, due to the small sample size of this pilot study, to ensure the preservation of participant anonymity.

### Data safety monitoring board (DSMB) and safety review plan

A Data Safety Monitoring Board (DSMB) will convene for the purposes of ensuring participant safety for the duration of the study. The DSMB will review the accumulated data for participant safety and AEs. The DSMB will meet following completion of the first participant, and quarterly thereafter. The DSMB comprises an Addiction Medicine specialist with expertise in the treatment of substance use disorders, a Senior Clinical Trialist with experience in the design and conduct of clinical trials, and a Biostatistician with experience in quantitative data analysis; all otherwise not involved in the study. The DSMB will make recommendations related to the continuation, modification or termination of the project as per the DSMB charter in line with NHMRC recommendations [56].

### COVID-19 trial modifications

As this clinical trial was developed during the COVID-19 pandemic, mitigating approaches were built into the protocol including pre-screening participants over the phone rather than in person and moving all follow-up visits to the participants’ choice of SMS or telephone contact to minimise potential SARS-CoV-2 exposure. As the trial is conducted in a large inner city public hospital it benefits from the hospital’s COVID-19 action plan, including (as indicated) screening of patients before entering a facility, temperature checks and the provision of personal protective equipment to all patients, clinicians and research staff. If the project is significantly impacted by COVID-19 (e.g. a major new outbreak) it will be the responsibility of the Principal Investigator to make any important modifications to the protocol as required to ensure participant safety, and to report according to new CONSORT and SPIRIT Extension for RCTs Revised in Extenuating Circumstances (CONSERVE) guidelines on the reporting of trials impacted by COVID-19 [57].

### Ethics and dissemination

This protocol (Version 3.0 dated 01 March 2021) was first approved by the St. Vincent’s Hospital Sydney Human Research Ethics Committee on 22 October 2020 (HREC approval no. 2020/ETH00683) and was prospectively registered with the Australia New Zealand Clinical Trials Registry (registration no. ACTRN12621000045819). Prior to participation, individuals are fully informed about the research and given the opportunity to enquire about details and decide whether or not to participate. If they agree to participate, they are asked to sign the consent form. A copy of the signed consent form is given to each participant. To ensure anonymity and to limit disclosure, participants are assigned a unique identifier at the time of enrolment. Results arising from the main study will be published in peer-reviewed journals and disseminated at international conferences. Results will be reported in such a manner that participants will not be identifiable.

## Discussion

To our knowledge this is the first trial of LDX for the treatment of MA withdrawal. Withdrawal is often the first stage of treatment for MAUD. Poorly managed symptoms of withdrawal may be a significant barrier to attaining treatment goals. LDX assisted withdrawal has the potential to improve treatment outcomes through mediating craving [39, 58, 59]. There is currently a paucity of research into treatments for MA withdrawal [10], with multiple sources noting that high quality research is urgently needed [15, 60, 61]. This study follows a novel approach to the management of acute stimulant withdrawal, similar to the management of opioid withdrawal with buprenorphine, which has proven effective in the past [21].

Limitations of this study include the lack of comparison group and small sample size; however, as this is the first time this medication has been used for this indication a single-arm un-blinded pilot study approach is appropriate.

Although not designed to detect efficacy, there are significant strengths to this study. The proposed tapering dose regimen may permit withdrawal symptoms and intensity of craving to be modulated over the acute withdrawal period, potentially assisting people to remain in withdrawal treatment for the duration of their care, providing more time for treatment planning and motivational enhancement. This study proposes a novel approach of commencing with a high dose stimulant more closely aligned with what would be consumed extra-medically by this population. Thus, we aim to reduce the risk of a Type-1 error due to under-dosing. Participants are closely monitored throughout the study period by specialist medical staff which will allow for high resolution safety data. This is further strengthened with a follow-up schedule to three weeks post discharge. The study protocol proposes medication delivery in a closely monitored inpatient withdrawal unit to accurately and safely document AEs, which is important as this medication has not been used in this context previously. Further, the proposed intervention is an add-on to usual care (the participants are not admitted expressly to be enrolled in the trial) increasing generalisability in real-world situations and enhancing relevance in other settings such as residential and outpatient services. Results from this study will provide valuable data for a planned RCT investigating the efficacy of LDX to treat MA withdrawal.

## Supporting information

S1 File SPIRIT checklist. (DOC)

S2 File Protocol approved by the ethics committee. (PDF)
