# Supplementary material for: Trial protocol of an open label pilot study of lisdexamfetamine for the treatment of acute methamphetamine withdrawal
Source: PLoS One. 2022 Oct 3;17(10):e0275371. doi: 10.1371/journal.pone.0275371 (PMC9529099; doi:10.1371/journal.pone.0275371)
Supplement: S2 File — (PDF) [file pone.0275371.s002.pdf]

An open-label trial of oral lisdexamfetamine for the treatment of acute methamphetamine withdrawal

OLAM Study

Sponsor: St. Vincent's Hospital Sydney

Version Control

1 March 2021 Version 1.3

Principal Investigator Protocol Agreement

I have read this protocol and I agree to conduct the study in accordance with the protocol and with all applicable government regulations including the Note for Guidance on Good Clinical Practice (CPMP/ICH/135/95) annotated with TGA comments (Australia, July 2000) and the National Health and Medical Research Council National Statement on Ethical Conduct in Human Research (2007-updated 2018).

Principal Investigator Name ____

Date (DD/MMM/YYYY) ____

PROJECT TEAM ROLES & RESPONSIBILITIES

Coordinating Principal Investigator: Krista Siefried

Organisation: UNSW

Department: National Centre for Clinical Research on Emerging Drugs

Position: Clinical Research Lead

Telephone no.: 9065 7808

Email: krista.siefried@svha.org.au

Responsibilities: Overall responsibility for the conduct of the study. Protocol development, data collection, analysis and dissemination. Supervision of Liam Acheson

Principal Investigator: Nadine Ezard

Organisation: SVHS / UNSW

Department: Alcohol and Drug Service / National Centre for Clinical Research on Emerging Drugs

Position: Clinical Director / Director

Telephone no.: 8382 1111

Email: nadine.ezard@svha.org.au

Responsibilities: Medical oversight of study. Principal site investigator. Protocol development, data analysis and interpretation and dissemination. Supervision of Liam Acheson

Investigator: Adrian Dunlop

Organisation: Hunter New England LHD

Department: Drug and Alcohol Clinical Services

Position: Director

Telephone no.: 1300 660 059

Email: adrian.dunlop@health.nsw.gov.au

Responsibilities: Protocol development, data interpretation, dissemination

Investigator: Nicholas Lintzeris

Organisation: South East Sydney LHD

Department: Drug and Alcohol Services

Position: Director

Telephone no.: 9332 8777

Email: Nicholas.lintzeris@health.nsw.gov.au

Responsibilities: Protocol development, data interpretation, dissemination

Co Investigator: Jonathan Brett

Organisation: SVHS

Department: Alcohol and Drug Service / Clinical Pharmacology and Toxicology

Position: Staff Specialist

Telephone no.: 8382 1111

Email: jonathan.brett@svha.org.au

Responsibilities: Protocol development, data analysis and interpretation and dissemination

Co Investigator: Liam Acheson

Organisation: SVHS / UNSW

Department: Alcohol and Drug Service / National Drug and Alcohol Research Centre

Position: Research Officer / PhD Candidate

Telephone no.: 8382 1666

Email: liam.acheson@svha.org.au

Responsibilities: Protocol development, data collection, analysis, interpretation and dissemination. Liam Acheson is a doctoral student under the supervision of Michael Farrell, Krista Siefried, Nadine Ezard and Rebecca McKetin.

Co Investigator: Craig Rodgers

Organisation: SVHS

Department: Alcohol and Drug Service

Position: Senior Staff Specialist

Telephone no.: 8382 1111

Email: craig.rogers@svha.org.au

Responsibilities: Protocol development, data interpretation and dissemination

Co Investigator: Anthony Gill

Organisation: SVHS

Department: Alcohol and Drug Service

Position: Clinical Director

Telephone no.: 8382 1111

Email: anthony.gill@svha.org.au

Responsibilities: Protocol development, data interpretation and dissemination

Co Investigator: Michael Farrell

Organisation: UNSW

Department: National Drug and Alcohol Research Centre

Position: Director

Telephone no.: 9385 0333

Email: michael.farrell@unsw.edu.au

Responsibilities: Protocol development, data interpretation and dissemination. Supervision of Liam Acheson

Co Investigator: Rebecca McKetin

Organisation: UNSW

Department: National Drug and Alcohol Research Centre

Position: Associate Professor

Telephone no.: 9385 0333

Email: r.mcketin@unsw.edu.au

Responsibilities: Protocol development, data interpretation and dissemination. Supervision of Liam Acheson

Co Investigator: Michael Christmass

Organisation: Next Step Drug and Alcohol Services

Department: Drug and Alcohol Services

Position: Consultant

Telephone no.: (08) 9219 1919

Email: michael.christmass@mhc.wa.gov.au

Responsibilities: Protocol development, data interpretation, dissemination

Co Investigator: Steve Shoptaw

Organisation: The University of California, Los Angeles

Department: Family Medicine / Psychiatry and Biobehavioural Sciences

Position: Professor

Telephone no.: +1 310 794 -0619 ext. 225

Email: sshoptaw@mednet.ucla.edu

Responsibilities: Protocol development, data interpretation, dissemination

Summary

**Study Title**	An open-label, safety and feasibility pilot study of tapering lisdexamfetamine for adult methamphetamine withdrawal inpatients
**Objectives**	Primary:<br>To determine the safety and feasibility of delivering a five-day tapering dose regimen of lisdexamfetamine (LDX) for the inpatient treatment of acute methamphetamine (MA) withdrawal.<br>Secondary:<br>• To describe the acceptability of a five-day tapering dose regimen of LDX for the treatment of acute MA withdrawal in treatment-seeking patients<br>• To describe participant retention to the study protocol for the duration of their withdrawal management during a five-day tapering dose regimen of LDX and ability to follow up post discharge<br>• To describe the changes in withdrawal severity and craving associated with a five-day tapering dose regimen of LDX in people acutely withdrawing from MA<br>• To describe the sleep-wake cycle of people with acute MA withdrawal in an inpatient withdrawal setting receiving a five-day tapering dose regimen of LDX
**Study design**	Open-label, single-arm, clinical trial
**Planned sample size**	n=15
**Selection criteria**	Inclusion Criteria:<br>• Adults over the age of 18 years<br>• Presenting to inpatient drug treatment services seeking treatment for acute MA withdrawal<br>• Methamphetamine use disorder as determined by an addiction medicine specialist according to the Diagnostic and Statistical Manual of Mental Disorders, 5th Edition (DSM-5) Criteria<br>• Last MA use within 72 hours of planned first study drug dose<br>• Have a positive urine drug screen for methamphetamines<br>• Willing and able to provide written informed consent and willingness to participate in and comply with the study<br>Exclusion Criteria:<br>• Women lactating, pregnant or of childbearing potential who are not willing to avoid becoming pregnant during the study

	• Expected concurrent withdrawal from alcohol, opioids, benzodiazepines, gamma-hydroxybutyrate or other gabapentinoids<br>• Known contradictions to lisdexamfetamine (as per product information, Appendix 1) including:<br>o Advanced arteriosclerosis<br>o Symptomatic cardiovascular disease including cardiac arrhythmia, ischaemic heart disease<br>o Moderate to severe hypertension<br>o Hyperthyroidism<br>o Known hypersensitivity or idiosyncratic reaction to sympathomimetic amines or any of the excipients<br>o Glaucoma<br>o Agitated states such as severe anxiety, tension and agitation<br>o During or within 14 days following the administration of monoamine oxidase inhibitors (hypertensive crises may result)<br>o Pheochromocytoma<br>o Tics, Tourette's syndrome<br>o Patients who currently exhibit severe depression, anorexia nervosa, psychotic symptoms or suicidal tendency<br>• Medically significant condition which in the opinion of a study medical officer renders a patient unsuitable for the study<br>• Involuntary patients (e.g. under the Mental Health Act in NSW)
**Study procedures**	In addition to standard of care inpatient withdrawal treatment participants will undergo the following study procedures:<br><br>Screening and Baseline<br><br>Admission, consent and formal screening procedures. Participants will complete the following questionnaires / surveys: Amphetamine Withdrawal Questionnaire (AWQ), Visual Analogue Scale (VAS), Wender-Utah Rating Scale (WURS), Columbia Suicide Severity Rating Scale (C-SSRS), Severity of Dependence Scale (SDS), Personal Wellbeing Index (PWI), Brief Psychotic Rating Scale (BPRS), ENRICHD Social Support Instrument (ESSI) and Timeline Follow Back (TLFB). Participants will undergo a, electrocardiogram (ECG) and full blood screen and urine drug screen (FBC, UEC, LFT).<br><br>Days 1-5<br><br>Participants will receive a tapering dose of oral lisdexamfetamine (250mg day 1, 200mg day 2, 150mg day 3, 100mg day 4, 50mg day 5) every morning as an inpatient. Participants will be required to complete the AWQ, VAS, Treatment Satisfaction Questionnaire for Medication TSQM II, BPRS (Day 5 only) and Consensus Sleep Diary

	(CSD) daily, and wear an actigraph (wearable sleep monitoring device) during their stay. Participants will be monitored for safety, concomitant medication and vital statistics daily.<br><br>Days 6-7<br><br>Days 6-7 will consist of two days inpatient monitoring following cessation of LDX. Participants will complete the AWQ, VAS, and CSD daily as before, and will continue to wear the actigraph. Participants will be monitored for safety, concomitant medication and vital statistics daily.<br><br>Days 14, 21 and 28<br><br>Days 14, 21 and 28 are for follow-up. These follow ups will be conducted with a study coordinator by telephone. Safety, concomitant medication, concomitant psychosocial therapy and ongoing care will be recorded. Participants will complete the BPRS at Day 7, and the AWQ, SDS, PWI and TLFB at Days 14, 21, 28.
**Statistical considerations**	Sample size calculation: N/A (pilot sample)<br><br>Analysis plan: Primary and secondary outcomes will be analysed using descriptive statistics. Continuous measures such as mean changes in AWQ and VAS craving scores from Baseline to Day 5 and Day 7 will be analysed. For categorical measures such as the presence of AEs, rates will be analysed using chi-square and relative risk.
**Study duration**	12 months
**ANZCTR Registration**	ACTRN12621000045819
**Sources of Financial Support**	Supported by the National Centre for Clinical Research on New and Emerging Drugs (NCCRED). NCCRED is funded by the Australian Government Department of Health
**Contact for public queries**	Mr Liam Acheson, 02 8382 1666, liam.acheson@svha.org,au
**Contact for scientific queries**	Dr Krista Siefried, 02 8382 1667, krista.siefried@svha.org.au
**Countries of recruitment**	Australia
**Health condition being studied**	Acute methamphetamine withdrawal

TABLE OF CONTENTS

1. BACKGROUND 10
1.1. METHAMPHETAMINE WITHDRAWAL 10
1.2. RATIONALE FOR PERFORMING THE STUDY 11
2. STUDY OBJECTIVES 12
2.1. PRIMARY OBJECTIVE 13
2.2. SECONDARY OBJECTIVES 13
2.3. OUTCOME MEASURES 13
3. STUDY DESIGN 14
3.1. DESIGN 14
3.2. STUDY GROUPS 14
3.3. NUMBER OF PARTICIPANTS 14
3.4. NUMBER OF SITES 14
3.5. DURATION 14
4. PARTICIPANT ELIGIBILITY 14
4.1. INCLUSION CRITERIA 14
4.2. EXCLUSION CRITERIA 14
5. STUDY OUTLINE 15
5.1. INVESTIGATION PLAN 15
5.2. RECRUITMENT AND SCREENING 17
5.3. INFORMED CONSENT PROCESS 17
5.4. ENROLMENT PROCEDURE 18
5.5. STUDY VISITS 18
5.5.1. Baseline Visit (Day 0) 18
5.5.2. Study Days 1-5 19
5.5.3. Days 6-7 19
5.5.4. Days 14, 21, and 28 19
5.6. SLEEP MEASURES 20
5.7. ADJUVANT SYMPTOMATIC MEDICATION 20
5.8. PARTICIPANT REIMBURSEMENT 20
5.9. STUDY DRUG AND PROCEDURE RISKS 21
6. TISSUE COLLECTION/BIOBANKING 22
7. SAFETY 22
7.1. ADVERSE EVENT REPORTING 23
7.2. ADVERSE EVENT 23
7.3. SERIOUS ADVERSE EVENT REPORTING 23
7.3.1. Serious adverse event (SAE): 23
7.3.2. Serious / Adverse Event Severity 24
7.3.3. Serious / Adverse Event Causality 24

7.3.4. **Serious / Adverse Event Expectedness** 25
7.4. EARLY TERMINATION 25
8. OUTCOMES AND FUTURE PLANS 25
9. STATISTICAL CONSIDERATIONS 25
10. CONFIDENTIALITY AND STORAGE AND ARCHIVING OF STUDY DOCUMENTS 26
11. PUBLICATIONS AND PRESENTATIONS 26
12. RESOURCES 27
13. APPENDIXES ERROR! BOOKMARK NOT DEFINED.
14. REFERENCES 27

1.BACKGROUND

1.1. METHAMPHETAMINE WITHDRAWAL

Australian Institute of Health and Welfare (AIHW) data list methamphetamine (MA) use second only to alcohol as the most common drug of concern in clients attending Alcohol and other Drug (AoD) services in Australia (1). While overall rates of MA use in Australia have remained stable in recent years, people who use MA are reporting higher rates of regular and dependent use (2). Daily and weekly use of meth/amphetamines among people who use MA have increased from 9.3% in 2010 to 20% in 2016 (3). In 2016, crystal methamphetamine ('ice') was the most commonly reported meth/amphetamine used, increasing from 22% in 2010 to 57% in 2016 (3). Australia has one of the highest documented rates of MA dependence in the world. It is estimated that 160,000 people were MA dependent in 2013/2014 (2). Beyond the individual, family, and community implications, this is estimated to cost Australia approximately $5 billion a year (4).

Cessation of stimulant use (including meth/amphetamines) among people who use stimulants regularly and chronically results in characteristic withdrawal symptoms (5). The DSM-5 lists the following symptoms in a stimulant withdrawal syndrome: dysphoria; fatigue; sleep disturbance; increased appetite; psychomotor agitation or retardation; and vivid dreams (6). Following and early "crash" phase (12-24 hours following last use), including exhaustion and fatigue (usually hypersomnia but sometimes insomnia or restless sleep), flat mood, anxiety, agitation, cravings and non-specific aches and pains; the profile of symptoms changes to more characteristic withdrawal symptoms including: strong cravings; mood fluctuations; irritability; restlessness; anxiety; agitation; fatigue; muscle tension; increased appetite; and poor concentration (5, 7, 8). Psychosis-like thought / perceptual disturbances may emerge, and chest pains and myocardial ischaemia have been reported. Peak withdrawal symptoms occur within the first 7 days, with symptoms persisting for 2-4 weeks (5). Supported withdrawal from MA is often the first step in a treatment journey, and furthermore, withdrawal completion is prerequisite for entering residential rehabilitation for those patients who wish to do so.

The acute MA withdrawal phase lasts 7-10 days (5). Importantly, no effective evidence-based treatment exists for methamphetamine withdrawal (9). Existing, consensus-based clinical guidelines incorporate a range of pharmacological approaches, but lack evidence of clinical efficacy in ameliorating symptom severity, duration of the withdrawal syndrome, or treatment retention. Current NSW guidelines were published in 2009 and have not been updated for over a decade. Unsuccessful MA withdrawal may result in important adverse outcomes including continued MA use, compromising individual attempts to reduce or cease use (10). Successful completion of the withdrawal phase can facilitate transition to ongoing AoD treatment, resulting in improved health and welfare (11, 12). Lack of effective treatment delays engagement with healthcare services (13), whilst early treatment engagement is associated with better substance use outcomes (14). Establishing effective, evidence-based approaches for the management of MA withdrawal is a critical priority for treatment of MA dependence.

A promising approach in the treatment of methamphetamine use disorder (MUD) is the use of lisdexamfetamine (LDX) to support patients in the acute withdrawal phase. LDX is currently under investigation as a longer-term treatment for MA dependence in a randomised controlled trial (RCT) (15). Substitution/replacement medication approaches have repeatedly been shown to assist treatment outcomes (e.g. nicotine replacement for tobacco/cigarettes; buprenorphine for opioids; nabiximols for cannabis) and a preliminary study of the safety of LDX in MA consuming adults also found that LDX suppresses MA cravings in people who use MA dependently (16).

1.2. Rationale for Performing the Study

There are no evidence-based pharmaceutical treatments for MA withdrawal (17). Ineffective treatment of withdrawal symptoms likely contribute to poor engagement in treatment and high rates of relapse as seen in the early post-MA-cessation period (first 6 months) (18). The lack of established, evidence-based treatment protocols for the management of MA withdrawal has been noted (9, 19, 20). One German review (19) and subsequent guidelines publication (9) noted that a review of Australian (as well US-American) clinical guidelines resulted in all of the guidelines being set aside, given they were "either not evidence-based or outdated by newer studies" (9). Based on the above, an effective and evidence-based treatment for MA withdrawal would theoretically improve retention early in treatment and hence provide better outcomes and reduce the risk of relapse compared to the currently available treatment options.

Measures of MA withdrawal, such as the Amphetamine Withdrawal Questionnaire (AWQ) (21); and MA craving, such as a visual analogue scale (VAS) (22), are subsequently related to level of substance use. Hence, a reduction of withdrawal and/or craving severity may predict a reduction in risk of relapse back to MA use in people withdrawing from MA.

Current treatment is mainly supportive. While psychosocial therapies (e.g. cognitive behaviour therapy or contingency management) have been associated with better outcomes (i.e. retention, abstinence) in people with MUD (23), these have not been demonstrated to be of value in the withdrawal setting. Current clinical practice is not well defined. It includes the use of symptomatic medications, usually short term benzodiazepines (e.g. diazepam) and/or antipsychotics (e.g. olanzapine) to manage agitation and irritability, despite an absence of evidence for their use in MA withdrawal (9).

The most recent Cochrane review of pharmacological treatment for amphetamine withdrawal found no effective pharmacotherapy for amphetamine or MA withdrawal (8). A more recent review of treatments for MA dependence noted that while there is no effective and accepted pharmacotherapy for MA dependence agonist therapies using a similar approach to opioid agonist therapy show promise and warrant further investigation (17). An often intended (or side) effect of MA consumption is decreased levels of sleep. Long term sleep deprivation has been associated with cognitive impairment, seizures and in extreme cases dementia and death. Importantly, both sleep deprivation and MA use are associated with psychotic symptoms (24-26). The link between sleep deprivation, MA use or withdrawal and psychosis has yet to be investigated in any serious capacity (27, 28). Previous studies have operated under laboratory conditions (29), or utilised questionnaires which may not give an accurate description of objective sleep parameters (30). To date no study has objectively measured sleep in people recently abstinent from MA, and the measurement of sleep wake cycles in this project will serve as a proof of concept for future studies.

In Australia, the clinical guidelines for MA withdrawal available in each State all reference the lack of evidence-based treatments. The most recent of these (Turning Point, Victoria, 2018) recommends symptomatic medications including: short-term use of tapering low-dose benzodiazepines (preferably diazepam) or atypical antipsychotics for pronounced agitation / insomnia; antipsychotic medication including haloperidol, chlorpromazine and atypical agents olanzapine or risperidone for psychotic features; symptomatic medications for other symptoms including non-specific (e.g. headache); selective serotonin reuptake inhibitors (SSRIs) for psychomotor slowing; magnesium or paracetamol for muscle aches / cramping. These recommendations differ very little from the NSW guideline (published in 2008) or the Queensland guidelines (published in 2012). The Drug and Alcohol Services South Australia (DASSA) guidelines are the only ones accompanied by an evidence report that provides medication dosing and frequency. These recommend supportive care and symptomatic medications including: mirtazapine for depression; short-term use of benzodiazepines (diazepam 5-10 mg BD prn) and antipsychotics (olanzapine 2.5-5mg BD prn) for a maximum of 7-10 days.

Neurobiological models of methamphetamine withdrawal suggest that the acute withdrawal syndrome may result from dopaminergic dysfunction related to chronic exposure to high dose amphetamines (31, 32). Medications to stabilise dopamine neurotransmission may therefore relieve the symptoms of the acute withdrawal syndrome (8). Dexamphetamine is a candidate medication, as it increases extracellular dopamine (33). Lisdexamfetamine (LDX), a pharmacologically inactive prodrug of dexamphetamine, is currently being examined in a randomised controlled trial (RCT) in the reduction in MA use (15).

LDX is absorbed after oral administration and hydrolysed to inactive metabolites and active dexamphetamine by red blood cells (34). LDX results in slower onset and lower peak concentration dopamine than dexamphetamine, allowing for once-daily administration and avoiding the rapid peak concentration observed with dexamphetamine administration. The plasma half-life of the active metabolite dexamphetamine is 10 hours (35), and steady state is achieved at Day 5 of once-daily LDX, there is no plasma accumulation of LDX (36). Further, LDX offers advantages over dexamphetamine in the treatment of MA withdrawal in the community, which is limited by concerns regarding abuse and diversion. Intravenous injection of LDX does not result in more rapid onset of action, and the blunted brain dopaminergic action reduces the positive reinforcing habit ("high"). As compared to dexamphetamine, LDX has less abuse-liking potential, and lower abuse potential (15, 37). Further, selecting the first 5 days of acute methamphetamine withdrawal aids in treating the inflammatory response to early stimulant withdrawal due to the oxidative nature of methamphetamine (38, 39), and to preserve cognitive function when it's most needed (i.e. to make ongoing treatment choices) (40, 41). Successful completion of the withdrawal phase can facilitate transition to ongoing AoD treatment and result in improved health and welfare (11, 12).

Our group (CIs Dunlop, Ezard, Lintzeris, Siefried) conducted a pilot study demonstrating the safety and tolerability of LDX in a sample of participants with MUD (16). Sixteen participants enrolled at two Australian AoD services in a dose-escalating, phase-2, open label, single-group study of oral LDX in adults with MA dependence. Eligibility included MA dependence for at least two years and reporting use of MA on at least 14 days of the preceding 28. A daily supervised dose of LDX was provided as a single-blinded ascending-descending dose regimen of 100 to 250mg oral LDX over 8 weeks. Of the 16 participants who enrolled, 14 (87.5%) completed to the highest dose of LDX (250mg) (Week 4). Participants responded to a visual analogue scale (VAS) for MA craving at baseline (no LDX), and then weekly at trough LDX concentrations. The mean VAS score at baseline was 57 (SD 32.8). At the 200mg LDX dose it was 40 (SD 24.3), and at 250mg decreased further to 33 (SD 24.8). While there was a mean difference in AWQ scores between baseline (mean 14.43 [SD 7.01]) and 250mg of LDX (12.64 [6.77]) these data are confounded due to participants' continued consumption of MA (i.e. not experiencing withdrawal). Importantly, there were no dose-limiting side effects or safety concerns. There are no studies of LDX for the treatment of MA withdrawal.

LDX may therefore be a candidate for medication assisted withdrawal in people who are dependent on methamphetamine, specifically people entering rehabilitation programs or those unwilling / unable to access maintenance programs (as no formal maintenance programs currently exist). There are no studies to date investigating LDX for the treatment of acute MA withdrawal.

2.STUDY OBJECTIVES

This study aims to assess the safety and feasibility of a tapering-dose regimen of LDX for the management of acute methamphetamine withdrawal in an inpatient detoxification setting. We hypothesise that a tapering dose of LDX is safe and feasible to deliver during routine inpatient withdrawal from methamphetamine.

2.1. PRIMARY OBJECTIVE

To determine the safety and feasibility of delivering a five-day tapering dose regimen of LDX for the inpatient treatment of acute methamphetamine withdrawal.

2.2. SECONDARY OBJECTIVES

- To describe the acceptability of a five-day tapering dose regimen of LDX for the treatment of acute MA withdrawal in treatment-seeking patients
- To describe participant retention to the study protocol for the duration of their withdrawal management during a five-day tapering dose regimen of LDX and ability to follow up post discharge
- To describe the changes in withdrawal severity and craving associated with a five-day tapering dose regimen of LDX in people acutely withdrawing from MA
- To describe the sleep-wake cycle of people with acute MA withdrawal in an inpatient withdrawal setting receiving a five-day tapering dose regimen of LDX

2.3. OUTCOME MEASURES

The measures used to assess the study outcomes are listed in Table 1.

Table 1 – Study outcomes and measures

Outcome	Measure
To determine the safety and feasibility of delivering a five-day tapering dose regimen of LDX for the inpatient treatment of acute methamphetamine withdrawal	Safety: Medical assessment, medical review (including biological parameters such as vital statistics and pathology results), development of serious/adverse events<br>Feasibility: Time taken to enrol sample and proportion of screen failures
To describe the acceptability of a five-day tapering dose regimen of LDX for the treatment of acute MA withdrawal in treatment seeking patients	Treatment Satisfaction Questionnaire for Medication (TSQM II)<br>Medication adherence (e.g. number of prescribed LDX doses taken)<br>Qualitative interviews<br>Adjuvant symptomatic medication log
To describe participant retention for the duration of their withdrawal management during a five-day tapering dose regimen of LDX and ability to follow up post discharge	Participant retention at Days 5 and 7 of inpatient period. Participant retention at Days 14, 21 and 28 of follow up period. Average number of days until participant discharge or withdrawal from study.
To describe the changes in withdrawal severity and craving associated with a five-day tapering dose regimen of LDX in people acutely withdrawing from MA	Change in subjective withdrawal severity (Amphetamine withdrawal questionnaire [AWQ]) and craving (Visual Analogue Scale]VAS]) over time.

To describe the sleep-wake cycle of people with acute MA withdrawal in an inpatient withdrawal setting receiving a five-day tapering dose regimen of LDX	Daily sleep diary (Consensus Sleep Diary [CSD]) and continuous actigraphy

3.STUDY DESIGN

3.1. DESIGN

Open-label, single-arm, pilot clinical trial.

3.2. STUDY GROUPS

This is a single group study of treatment seeking adults admitted to an acute inpatient withdrawal unit for MA withdrawal.

3.3. NUMBER OF PARTICIPANTS

15 participants are to be recruited at St. Vincent's Hospital, Sydney (SVHS) (NSW), Australia.

3.4. NUMBER OF SITES

This is a single site study at SVHS.

3.5. DURATION

Participant recruitment will commence following HREC approval, site authorisation, and associated study set-up, and will continue with data collection over a period of 6 months. The study duration will be 12 months to allow for study approvals and set-up, study reporting, results dissemination, and study close-out.

4. PARTICIPANT ELIGIBILITY

4.1. INCLUSION CRITERIA

- Adults over the age of 18 years
- Presenting to inpatient drug treatment services seeking treatment for MA withdrawal
- Methamphetamine use disorder as determined by an addiction medicine specialist according to the Diagnostic and Statistical Manual of Mental Disorders, 5th Edition (DSM-5) Criteria (6)
- Last MA use within 72 hours of planned first study drug dose
- Have a positive urine drug screen for methamphetamines
- Willing and able to provide written informed consent and willingness to participate in and comply with the study

4.2. EXCLUSION CRITERIA

- Lactating, pregnant or of childbearing potential and not willing to avoid becoming pregnant during the study
- Expected concurrent withdrawal from alcohol, opioids, benzodiazepines, gamma-hydroxybutyrate or other gabapentinoids
- Known contradictions to lisdexamfetamine (as per product information, Appendix 1) including:

- Advanced arteriosclerosis
- Symptomatic cardiovascular disease including cardiac arrhythmia, ischaemic heart disease
- Moderate to severe hypertension
- Hyperthyroidism
- Known hypersensitivity or idiosyncratic reaction to sympathomimetic amines or any of the excipients
- Glaucoma
- Agitated states such as severe anxiety, tension and agitation
- During or within 14 days following the administration of monoamine oxidase inhibitors (hypertensive crises may result)
- Pheochromocytoma
- Tics, Tourette's syndrome
- Patients who currently exhibit severe depression, anorexia nervosa, psychotic symptoms or suicidal tendency

- Medically significant condition which in the opinion of a study medical officer renders a patient unsuitable for the study
- Involuntary patients (e.g. under the Mental Health Act 2007, NSW)

5. STUDY OUTLINE

5.1. INVESTIGATION PLAN

Table 2: Schedule of Assessments

Assessment / intervention	Study Day										
	Sc[a]	1	2	3	4	5	6	7	14	21	28
Screening / eligibility											
Consent	•										
Eligibility checklist	•										
hCG dipstick[b]	•										
Urine Drug Screen – dipstick	•										
Safety											
Medical assessment (current SUD, prior treatment, medical and psychiatric history, concomitant medication, comorbidities)	☐	☐	☐	☐	☐	☐	☐	☐			☐
Medical review (safety): (Blood Pressure, Heart Rate, Temperature, RR, SpO_2 (Once at screening, QID days 1-	☐	☐	☐	☐	☐	☐	☐	☐			☐

7 as per standard of care)											
Suicidality - C-SSRS	•					◆			◆		
12 lead electrocardiogram (ECG)	•	○	○	○	○	○	○	○			
Adverse events	•	•	•	•	•	•	•	•	•	•	•
Demographics											
Demographics (including lifetime and recent substance use; SDS)	•										
Study Procedures											
Standard of care pathology (FBC, UEC, LFT)	□										
ADHD screening - WURS	•										
Quality of Life – PWI	•								•	•	•
Social Support – ESSI	•										
Study drug		•	•	•	•	•					
Urine drug screen - urinalysis	□										
Concomitant medication record in the prior 24 hours (days 1-7) / 7 days (day 14, 21 28) (over the counter and prescribed medication including NRT)	•	•	•	•	•	•	•	•	•	•	•
Withdrawal – AWQ	•	•	•	•	•	•	•	•	•	•	•
Craving – VAS	•	•	•	•	•	•	•	•			
Substance use - TLFB	•								•	•	•
Substance use in last 24 hours		•	•	•	•	•	•	•			
Treatment satisfaction – TSQM-2		•	•	•	•	•					
Sleep diary – CSD modified		•	•	•	•	•	•	•			
Objective sleep measure - actigraphy		•	•	•	•	•	•	•			
Qualitative interview				◄	─	─	─	►			
Psychosis – BPRS	•					•			•		
Dependence – SDS	•								•	•	•
Record of ongoing / engagement in care (i.e. one on one									•	•	•

counselling, group work, residential rehab, NA/AA)											
Participant reimbursement	•					•		•	•	•	•

Grey = inpatient period • = study activity ☐ = standard of care ⟷ = study activity conducted any time on indicated days inclusive ○ = if clinically indicated ◆ = follow up version of measure
[a] screening (may be completed prior to admission if convenient to participant and researcher. If conducted prior to admission hCG and UDS *must* only be completed on admission [b] people of childbearing potential only

5.2. Recruitment and Screening

Participants will be recruited via the SVHS AoD Specialist Treatment Services. Procedures for our group's (CI Dunlop, Ezard, Lintzeris, Siefried) current study of LDX for the treatment of methamphetamine dependence (NHMRC APP1109466) will be utilised. During the study recruitment period, treatment-seeking patients presenting for inpatient withdrawal who are broadly eligible for the study will be approached by study staff to discuss the study before they are invited to undergo screening for eligibility.

Pre-screening of broadly eligible patients (i.e. those admitted or referred for inpatient acute methamphetamine withdrawal) will be completed by the Research Officer or Clinical Trials Nurse either in person or by telephone. A pre-screening log will be maintained by study staff at the study site. Potentially eligible participants will be invited to a screening assessment. If a patient consents, screening procedures may begin prior to admission during an additional visit to the hospital (e.g. for a planned withdrawal inpatient admission).

De-identified demographic and basic clinical information relating to each patient who is screened for eligibility will be maintained by study staff. This will be captured on the re-identifiable screening log and will be collected irrespective of if the patient is eligible or proceeds to enrolment. If the participant is ineligible or elects not to proceed to study enrolment the reason(s) for this will be recorded.

Screening will continue until the target sample is achieved.

Re-identifiable screening logs for this study will be generated by the Research Officer.

Screen failures will be collated and reported in study outputs to demonstrate the study's ability to represent the underlying population.

5.3. Informed Consent Process

Patients who are interested in study participation will be consented to the study and assessed for medical eligibility by a study investigator or delegated Addiction Medicine Specialist.

Patients will be given the current Human Research Ethics Committee (HREC) approved participant information sheet for their consideration.

The investigator, or a person designated by the investigator, will fully inform the patient of all pertinent aspects of the study. The patient shall be given ample time and opportunity to inquire about details of the study, to discuss the study with others, and to decide whether or not to participate in the study. All questions about the study will be answered to the satisfaction of the patient. To avoid possible coercion, the investigator or delegate who obtains consent will not be directly engaged in the participants care.

Prior to a patient's participation in the trial, the written informed consent form will be signed and personally dated by the participant and by the person who conducted the informed consent discussion.

If a participant is unable to read, then an impartial witness will be present during the entire informed consent discussion and will sign and date the consent form to attest that the information in the consent form and any other written information was accurately explained to, and apparently understood by, the participant.

Screening for eligibility will include medical review by an Addiction Medicine Specialist and Human chorionic gonadotrophin (hCG) blood test for pregnancy. Participants who meet all eligibility criteria will be enrolled into the study and provided a unique study identification number.

5.4. Enrolment Procedure

Following informed consent procedures, participants will be enrolled into the study and entered into the enrolment log, which will provide a unique participant study identification number.

If the time between enrolment and first dose of study drug exceeds 14 days (i.e., if a participant is enrolled in the study but decides to delay hospital admission), the screening for eligibility may be required to be repeated. This will be determined on a case-by-case basis by the Co-ordinating Principal Investigator.

5.5. Study Visits

5.5.1. Baseline Visit (Day 0)

The screening and baseline visits may be combined for inpatient admissions (e.g. transferred from the emergency department to the inpatient withdrawal unit) or may be commenced prior to admission for those participants with planned admissions. Participants may be pre-screened via the telephone any time prior to admission if admission is planned. Participants will be provided with a Participant Information Sheet prior to admission and encouraged to discuss participation with their friends, family and general practitioner. Formal eligibility screening, consent procedures and enrolment will commence upon admission.

Upon admission and following the informed consent procedures a study coordinator will collect baseline data including: demographics; medical history; concomitant medications; lifetime and recent substance use; prior treatment history. Patient medical records will be accessed as part of their medical history assessment to determine past and current human immunodeficiency virus (HIV) and hepatitis C infection. Study participants will complete the Columbia Suicide Severity Rating Scale (C-SSRS) (42), Wender-Utah Rating Scale (WURS) (43) Severity of Dependence Scale (SDS) (44), AWQ (21), visual analogue scale (VAS) (22), Brief Psychiatric Rating Scale (BPRS) items of unusual thought content, hallucinations and suspiciousness (45, 46), Personal Wellbeing Index (PWI) (47), ENRICHD Social Support Inventory (ESSI) (48) and timeline follow back (one month at screening, 7 days at Days 14, 21 and 28) (TLFB) (49). A dipstick urine drug screen and formal urinalysis will be collected to verify self-reported drug use. Standard care blood tests will be collected, including: full blood count (FBC); urine/electrolytes/creatinine (UEC); liver function tests (LFT); +/- HIV and Hepatitis C serology (based on clinical judgement as per standard of care).

Participants may be screened for eligibility in person on the day of admission if their admission is not planned.

5.5.2. Study Days 1-5

Day 1 of the study treatment period will be the first day following inpatient admission for acute MA withdrawal. Participants will receive their first dose of study drug on the first morning available 24-48 hours post last MA use. If unfeasible in desired dosing window patients may receive the study drug <24 hours post use or 24-72 hours post use.

Participants will complete the AWQ, VAS (22), TQSM (50), and a modified Consensus Sleep Diary (CSD) (51), daily. Additionally, participants will be required to wear an actigraph for the duration of their inpatient stay (see below for details on sleep measures). Participants will complete the BPRS on Day 5 only. Participants will be assessed daily for concomitant medications, and concomitant psychosocial therapies. Study drug and questionnaires should be administered daily within a 1-hour (+/-) window of time, within 4 hours of waking each morning. Participants will receive a tapering dose of LDX with 250mg of LDX on Day 1, tapering by 50mg/day to 50mg on Day 5. A dosing schedule is provided in **Table 3**

Participants will be assessed daily for safety assessments including: adverse events (AEs); vital signs (blood pressure, temperature, heart rate, respiratory rate, oxygen saturation) will be measured four times a day (at 6am, 10 am, 4pm and 8pm).

Adjuvant symptomatic medication (diazepam +/- olanzapine) may be provided based on the clinical judgement of the treating Addiction Medicine Specialist (see below).

Table 3: Dosing schedule

Study day	Day 1	Day 2	Day 3	Day 4	Day 5	Day 6	Day 7
lisdexamfetamine	250 mg OD[a]	200 mg OD	150 mg OD	100 mg OD	50 mg OD	-	-

[a] OD, daily; dosing should be within +/- 1 hour of scheduled dosing time

5.5.3. Study Days 6-7

Days 6-7 will consist of two days inpatient monitoring following cessation of lisdexamphetamine for withdrawal symptoms and adverse events, as well as for ongoing inpatient treatment and recovery planning. These two days allow a participant to be stimulant free upon discharge for admission to a rehabilitation centre if required. Participants will complete the AWQ, VAS, and CSD daily as before, and will continue to wear the actigraph Adjuvant symptomatic medication (diazepam +/- olanzapine) may continue based on the clinical judgement of the treating addiction medicine specialist. Participants will be assessed daily for AEs and concomitant medications, and any psychosocial therapies received will be recorded. Further inpatient hospitalisation following Day 7 will be at the treating clinicians' discretion and will be independent of the study.

A discharge medical review will be conducted on Day 7 (or earlier if a participant withdraws from the study). Participants will discharge from inpatient care at Day 7 unless there are other clinical indications (e.g. comorbidities) or discharge planning requiring ongoing inpatient care.

5.5.4. Study Days 14, 21, and 28

Participants will receive telephone follow-up and short message service (SMS) from a study coordinator. Safety, concomitant medication, concomitant psychosocial therapy and ongoing care will be recorded. Participants will complete the BPRS at Day 7, and the AWQ, SDS, PWI and TLFB at Days 14, 21, 28. Participants will have the option to complete questionnaires using a smartphone application. The full schedule of assessments is available in **Table 2**.

5.6. Sleep measures

Reliable ambulatory monitoring of sleep quality involves a combination of qualitative and quantitative measures. Participants will be required to complete a modified Consensus Sleep Diary (CSD) (51) daily, within one hour of waking to collect qualitative experiences of sleep quality. Participants will also be provided with a Phillips actigraph on Day 1, and will be required to wear it for the duration of their inpatient stay. This device is non-invasive and appears similar to a wristwatch without a clockface, and continuously collects data on the wearers level of movement, body temperature and ambient light levels. The device is water resistant and may be worn in the shower, however participants may elect to remove the device to shower if they wish. Participants will be required to return the device before discharge from the unit.

As the Gorman Unit is a "lockdown" ward which does not permit re-entry, if a participant decided to self-discharge the risk of someone absconding unexpectedly with the device is very low.

5.7. Qualitative interviews

Participants will be invited to participate in a qualitative interview to examine their experiences of participating in the clinical trial to ensure any subsequent trials will include participant perspectives. Interviews will be semi-structured, one-on-one interviews of approximately 30 minutes, examining the themes of expectation and expectation management, experience of participating in the trial, thoughts on how the trial could be improved in the future and general concerns around staying in hospital, medication requirements and how the potential of a placebo may change their perspectives. Participating in the interview is not a requirement to participate in the full study, and participants will have the option to opt-into this part of the study during the informed consent process.

All interviews will be conducted by study staff trained in qualitative interviewing, at a mutually suitable time for researcher and participant between Days 3 and 7 of the inpatient admission. All interviews will be conducted in a private interview room and audio recorded. Recordings will be transcribed by an independent transcriber and cleaned by the researcher who conducted the interviews prior to analysis.

5.8. Adjuvant Symptomatic Medication

Adjuvant symptomatic medication will be provided to patients who display severe sleep disturbance, anxiety or psychotic symptoms as required as per standard of care. Patients may be provided with 5-10mg of diazepam up to four times daily (QID) and/or 2.5-5mg olanzapine three times daily (TDS) as prescribed by a medical officer as per *SVHS Management of Methamphetamine Use Disorder Procedure* (Appendix 2). If a participant requires more symptomatic medication than protocoled above the participant will undergo a medical review by PI Ezard. This may then be recorded as an adverse event, and a participant may cease to receive the study drug if required based on clinical judgement. Participants may remain enrolled in trial while not receiving the medication to complete measures of withdrawal and craving unless they elect to be withdrawn from the study entirely. These data will be collated and reported in study outputs to demonstrate feasibility of the study drug.

5.9. Participant reimbursement

Participants who consent to partake in the study and attend at least one day of inpatient withdrawal management will receive a $20 grocery gift card. If a patient remains in treatment until Day 5, they

will receive an additional $30 gift card, and if they remain in treatment until Day 7, they will receive a second $30 gift card. Participants will also be reimbursed $30 for each follow up appointment (either telephone or face-to-face, up to a maximum of three) that they attend. Total possible reimbursement for participation in this study is $170 and is described in Table 4

Table 4: Reimbursement schedule

Study day	Day 0	Day 5	Day 7	Day 14	Day 21	Day 28
Reimbursement amount	$20	$30	$30	$30	$30	$30

5.10. STUDY DRUG AND PROCEDURE RISKS

All medications have risks of side effects. Many of the side effects that have been associated with lisdexamfetamine are also seen in methamphetamine use. **Table 5** reports adverse drug reaction rates in lisdexamfetamine compared to placebo in short-term, parallel-group, controlled studies in adults. This has been adapted from the Australian Therapeutic Goods Administration (TGA) (52).

Table 5 – Reactions occurring in ≥5% of adults

Preferred Term	Vyvanse N=493 (n [%])	Placebo N=202 (n [%])
Decreased appetite	122 (24.7)	6 (3.0)
Dry mouth	113 (22.9)	8 (4.0)
Insomnia	79 (16.0)	12 (5.9)
Headache	100 (20.3)	13 (6.4)
Irritability	31 (6.3)	7 (3.5)
Anxiety	25 (5.1)	0
Nausea	26 (5.3)	5 (2.5)
Fatigue	25 (5.1)	7 (3.5)
Weight decreased	19 (3.9)	0
Diarrhoea	29 (5.9)	2 (1.0)
Feeling jittery	25 (5.1)	0
Initial insomnia	26 (5.3)	6 (3.0)

Adverse Drug Reactions occurring in ≥5% of adults who received Vyvanse in short-term, parallel-group, controlled studies.

Medications in this drug class have also been associated with the following side effects:

- Rash and fever
- Shortness of breath, excessive sweating, and excessive widening of the pupil
- Decreased sex drive and erectile dysfunction
- Excessive motor activity with or without feelings of restlessness
- Tic, tremor
- Rapid heartbeat (tachycardia), palpitations, increased blood pressure

Lisdexamfetamine is traded under the name Vyvanse® and is manufactured by Takeda Pharmaceutical Company Ltd. Vyvanse is indicated for the treatment of attention deficit hyperactivity disorder (ADHD) and binge eating disorder in Australia. Vyvanse capsules contain 20mg, 30mg, 40mg, 50mg, 60mg or 70mg of lisdexamfetamine dimesilate and was developed for once-daily oral administration.

Lisdexamfetamine is an inactive prodrug of dexamphetamine. After oral administration, lisdexamfetamine is rapidly absorbed from the gastrointestinal tract and hydrolysed primarily in whole blood to dexamphetamine. Amphetamines are non-catecholamine sympathomimetic amines with CNS stimulant activity (53).

This study proposes a novel tapering dosing regimen starting at a higher dose than indicated for other medical conditions (ADHD, binge-eating disorder). Previous yet unpublished research by this group (CI's Dunlop, Ezard, Lintzeris, Siefried) has demonstrated safety of doses up to 250mg in a sample of people dependent on MA (16), and a study investigating 250mg oral lisdexamfetamine daily in people with schizophrenia found no drug-related serious adverse events (54)

Approximately 96% of lisdexamfetamine is excreted in urine and 0.3% through faeces over a period of 120 hours. The plasma elimination half-life of lisdexamfetamine typically averages less than 1 hour (52).

As this is an open-label study protocol, the lisdexamfetamine will be sourced from the SVHS pharmacy. Study drug will be prescribed by an investigator overseeing the participants inpatient care (CI Brett, Ezard, Gill, Rodgers). The study medication will be transported to the Alcohol and Drug Service inpatient withdrawal unit medication safe, and administered by inpatient registered nursing staff or a clinical trial registered nurse.

Lisdexamfetamine will not be available following the final dose of study drug. Ongoing standard of care treatment will be individualised to the participant and in collaboration with their treating clinicians.

6. TISSUE COLLECTION/BIOBANKING

This study examines a tapering dose of lisdexamfetamine in addition to treatment as usual in a population of adults being treated for MA withdrawal in an inpatient setting. As such, participants will continue to receive treatment and testing that is usually administered as standard of care. Blood and urine tests will be conducted as part of routine clinical care for participants enrolled in the study. Blood taken will undergo testing for full blood count (FBC), urine/electrolytes/creatinine (UEC), liver function tests (LFTs) and HIV and/or Hepatitis C serology based on clinical judgement. All tests are part of routine care and will be analysed by Sydney Pathology (SydPath), SVHS, and will be destroyed post analysis. Urine collected for dipstick tests is not standard of care, and will be destroyed post qualitative analysis by the investigators. One urine sample from each participant (Screening) will be sent to Sydpath for analysis as per standard of care.

Samples will not be stored, transported or banked.

7.SAFETY

Safety has been defined as any untoward medical occurrence that results in the following: death, is life-threatening, requires inpatient hospitalisation or prolongation of existing hospitalisation, persistent or significant disability/incapacity or congenital/birth defect, condition requiring medical or surgical intervention. An adverse event can therefore be any unfavourable or unintended sign, symptom or condition and/or an observation that may or may not be related to the study treatment.

Safety will be monitored continuously through the inpatient stage of the trial with regular nursing and medical assessment, with oversite from each sites PI. Follow-up safety will be assessed at Days

14, 21 and 28 by telephone or in person. Safety data will be collected through Adverse Event and Serious Adverse Event reporting.

7.1. Adverse Event Reporting

7.2. Adverse event

An Adverse Event (AE) is also referred to as an adverse experience. An AE is any untoward medical occurrence in a patient or clinical trial participant administered a pharmaceutical product and which does not necessarily have a causal relationship with this treatment.

An adverse event can therefore be any unfavourable and unintended sign, symptom, or disease temporally associated with the use of an investigational medicinal product (IMP), whether or not related to the IMP.

An Adverse Reaction (AR) is any untoward and unintended response to an IMP related to any dose administered. This means that any AE judged by the reporting PI or the sponsor as having a reasonable causal relationship (i.e. evidence to argue or suggest a causal relationship) to an IMP would qualify as an AR.

Each AE or AR must be evaluated by the PI for:

- Seriousness: An assessment of whether the AE meets the definition of an SAE
- Severity: A grading of the severity of the AE
- Causality: A clinical assessment of whether there is a reasonable causal relationship between the adverse event and the IMP
- Expectedness: An assessment of whether the adverse reaction is consistent with the information previously described in the product label

7.3. Serious Adverse Event Reporting

7.3.1. Serious adverse event (SAE):

For medicines, also referred to as serious adverse drug reaction, any untoward medical occurrence that at any dose:

- results in death;
- is life-threatening;
- requires in-patient hospitalisation or prolongation of existing hospitalisation;
- results in persistent or significant disability/incapacity;
- is a congenital anomaly/birth defect; or
- is a medically important event or reaction.

NOTE: The term 'life-threatening' in the definition of 'serious' refers to an event in which the patient was at risk of death at the time of the event; it does not refer to an event/reaction which hypothetically might have caused death if it were more severe.

The coordinating principal investigator and/or the research officer must be notified within 24 hours regarding the occurrence of any SAE. The coordinating principal investigator and/or the research officer will report any SAE that fulfils the criteria for expedited reporting (i.e. unexpected and drug related events) to the appropriate regulatory authorities within the required reporting time frame.

SAEs must be reported to the lead approving ethics committee (SVHS HREC) in real time if they meet the criteria of a Significant Safety Issue (SSI) as defined by the Office for Health and Medical Research (OHMR) in New South Wales. The research officer under the supervision of the coordinating principal investigator will be responsible for reporting all SAEs to pharmaceutical companies as / if necessary.

Any SAE that is ongoing at the final study visit must be followed until resolution or until the event stabilises (for those events that will not resolve).

7.3.2. Serious / Adverse Event Severity

Grading of severity of AE's and SAE's will follow the US Department of Health and Human Services (DHHS) National Institutes of Health (NIH) Division of AIDS (DAIDS) Table for grading the severity of adults and paediatric adverse events (55). The grading table provides a scale ranging from Grade 1 through to Grade 5, under the following general guidelines:

- Grade 1 – indicates a mild event
- Grade 2 – indicates a moderate event
- Grade 3 – indicates a severe event
- Grade 4 – indicates a potentially life-threatening event
- Grade 5 – indicates death

If the severity of an AE could fall into either one of two grades (i.e., the severity of the AE could be either Grade 2 or Grade 3), sites should select the *higher* of the two grades. When determining the severity grade, guidance provided in Table 6 should be considered.

Table 6 – Estimating the severity grade of AEs/SAEs

Grade 1 – Mild	Grade 2 - Moderate	Grade 3 –Severe	Grade 4 – Potentially life-threatening
Mild symptoms causing no or minimal interference with usual social and / or functional activities with no intervention indicated	Moderate symptoms causing greater than minimal interference with usual social and / or functional activities with intervention indicated	Severe symptoms causing inability to perform usual social and / or functional activities with intervention or hospitalisation indicated	Potentially life-threatening symptoms causing inability to perform basic self-care functions with intervention indicated to prevent permanent impairment, persistent disability, or death

Adapted from the US DHHS NIH DAIDS Table for Grading the Severity of AEs

7.3.3. Serious / Adverse Event Causality

For each AE/SAE, the PI must make a clinical assessment of whether there is reasonable causal relationship between the AE/SAE and the investigational medicinal product. This study will adhere to the Council of International Organisations of Medical Sciences (CIOMS) VI Working Group recommendation that the PI provide a simple binary decision for drug causality, that is: related or not related (56).

7.3.4. Serious / Adverse Event Expectedness

For each Adverse Event, the PI must make an assessment of whether the AE/SAE is consistent with the information previously described in the investigational medicinal products TGA approved product label.

The PI will assess the AE/SAE as a Suspected Unexpected Serious Adverse Reaction (SUSAR) if the event is a suspected adverse reaction that is both serious and unexpected. That is, a suspected adverse drug reaction is considered “unexpected” if it is not listed in the product label, or it is not listed at the specificity or severity that has been observed. This also refers to AEs or ARs that are mentioned in the product label as occurring with a class of drugs or anticipated from the pharmacological properties of the drug, but are not specifically mentioned as occurring with the IMP. Seriousness is determined as above.

7.4. Early Termination

This study may be terminated early if there is a serious breach of confidentiality or safety event. The CPI is responsible for determining if the study needs to be terminated, and for informing participants, correspondence to HREC and compiling a final study report.

8.DATA SAFETY MONITORING BOARD

A DSMB will be established prior to study recruitment, and the DSMB membership will include: an Addiction Medicine Specialist; a Psychologist; and a Biostatistician (all not otherwise involved with the study). The DSMB will convene following first participant, first visit, and quarterly thereafter. All Serious Adverse Events (SAEs) and Suspected Unexpected Serious Adverse Reactions (SUSARs) will be reviewed by the DSMB quarterly. Following each meeting, the DSMB will advise one of four options: continue study as per protocol, continue study with protocol amendments, suspend study, or discontinue study. Study suspension or discontinuation will be based on pre-determined safety criteria.

9.OUTCOMES AND FUTURE PLANS

Results from this pilot study will be used in the development of a fully powered, multi-site, randomised controlled trial investigating lisdexamfetamine for the treatment of acute methamphetamine withdrawal.

Results from this study will also be published in peer-reviewed journals and presented at national and international conferences and disseminated in AI Acheson’s doctoral thesis.

10.STATISTICAL CONSIDERATIONS

This is a pilot study and as such no power calculations have been completed. The study will enrol 15 participants, conventional for an open-label single-arm study (57, 58).

Primary and secondary outcomes will be analysed using descriptive statistics. Continuous measures such as mean changes in AWQ and VAS craving scores from Baseline to Day 5 and Day 7 will be analysed. For categorical measures such as the presence of AEs, rates will be analysed using chi-square and relative risk.

Qualitative interviews will be collected until all trial participants have been approached. Interviews will be thematically analysed to extract key themes across the responses.

11.CONFIDENTIALITY AND STORAGE AND ARCHIVING OF STUDY DOCUMENTS

This study will use electronic data capture in the form of REDCap (Research Electronic Data Capture). All data entered to REDCap will be unidentifiable as it will be entered via the unique study identification number assigned at enrollment to the study. The REDCap database is located on a standalone database server hosted by St Vincent's Hospital Sydney (SVHS). The database server resides behind the SVHS internal firewall and access to the server is controlled via firewall rules. All data collected via REDCap is backed up daily, both on the local server and by the SVHS backup system. All connections to the system, both external and internal, will occur over encrypted channels.

Access to study records within the database will be limited by using Data Access Groups (DAGS). Only users within a given DAGs can access records created by users within that group. Access to components of study records is role-based and can only be granted by the Research Officer or CPI. All data entered into REDCap for the purpose of data analyses will be de-identified and traceable to supporting identifiable source documentation such as hospital/medical records (including electronic health records), laboratory results, data recorded in automated instruments and pharmacy records, etc. only by the study team by accessing the re-identifiable study logs which will be stored and maintained independently of the REDCap database.

All electronic identifiable / re-identifiable study data will be stored in a password protected document on a secure, internal server. Physical data will be stored in filing cabinets or folders within the relevant departments accessible to study staff in locked units or swipe card accessible departments. Only PIs or delegated study staff will be permitted to access the data. All source documentation will be held confidentially in line with current legislation governing health information, and will not be made publicly available.

Following the conclusion of the study both physical and digital records will be archived for a period of 15 years as per NHMRC and ICH-GCP guidelines (59). After the archiving period has lapsed physical data will be incinerated and digital data permanently deleted from hard drives.
The site Principal Investigator (PI) is responsible for study documentation and archiving. Should the site PI retire, relocate, or for other reasons withdraw from the responsibility of keeping the study records, custody will be transferred to the replacement site PI.

12.PUBLICATIONS AND PRESENTATIONS

The Coordinating Principal Investigator (CPI) and Study Investigator Team are responsible for the presentations and publications arising from this study. All potential or suggested publications and presentations are required to go through the CPI and Study Investigator Team and will be included on a formalised publication policy and plan. The main findings will be published as a thesis chapter (L Acheson).

13.RESOURCES

This project is supported by the National Centre for Clinical Research on Emerging Drugs (NCCRED). NCCRED is funded by the Australian Government Department of Health. The funding body has no role in the study design, collection, management, analysis and interpretation of the data, writing of the report, and the decision to submit for publication.

14. APPENDICES

Appendix 1: Australian Product Information – lisdexamfetamine (Attached and available from https://www.ebs.tga.gov.au/ebs/picmi/picmirepository.nsf/pdf?OpenAgent&id=CP-2013-PI-02051-1&d=202007301016933)

Appendix 2: Management of Methamphetamine Use Disorder (SVHS Policy) (Attached)

Appendix 3: Product Information – Phillips actiwatch 2 Wearer Guide (Attached)

Appendix 4: Product Information - Phillips actiwatch 2 Clinician Guide (Attached)

Appendix 5: Product Information - Phillips actiwatch 2 Technician Guide (Attached)

Appendix 6: OLAM Interview Schedule

15.REFERENCES

1. AIHW. Alcohol and other drug treatment services in Australia 2017–18: key findings. Canberra: AIHW; 2019.

2. Degenhardt L, Larney S, Chan G, Dobbins T, Weier M, Roxburgh A, et al. Estimating the number of regular and dependent methamphetamine users in Australia, 2002–2014. Med J Aust. 2016;204(4):153-.

3. AIHW. National drug strategy household survey 2016: Detailed findings. Canberra, Australia, 2017.

4. Whetton S, Shanahan M, Cartwright K, Duraisingam V, Ferrante A, Gray D, et al. The social costs of methamphetamine in Australia 2013/14. Tait RJ, Allsop S, editors. Perth, Western Australia: National Drug Research Institute, Curtin University; 2016.

5. McGregor C, Srisurapanont M, Jittiwutikarn J, Laobhripatr S, Wongtan T, White JM. The nature, time course and severity of methamphetamine withdrawal. Addiction. 2005;100(9):1320-9.

6. Diagnostic and statistical manual of mental disorders : DSM-5. Fifth edition. ed. Arlington, VA: American Psychiatric Publishing; 2013.

7. Newton TF, Kalechstein AD, Duran S, Vansluis N, Ling W. Methamphetamine abstinence syndrome: preliminary findings. Am J Addict. 2004;13(3):248-55.

8. Shoptaw SJ, Kao U, Heinzerling K, Ling W. Treatment for amphetamine withdrawal. Cochrane. 2009(2).

9. Hartel-Petri R, Krampe-Scheidler A, Braunwarth WD, Havemann-Reinecke U, Jeschke P, Looser W, et al. Evidence-Based Guidelines for the Pharmacologic Management of Methamphetamine Dependence, Relapse Prevention, Chronic Methamphetamine-Related, and Comorbid Psychiatric Disorders in Post-Acute Settings. Pharmacopsychiatry. 2017;50(3):96-104.

10. Hartz DT, Frederick-Osborne SL, Galloway GP. Craving predicts use during treatment for methamphetamine dependence: a prospective, repeated-measures, within-subject analysis. DrugAlcohol Depend. 2001;63(3):269-76.

11. Ford LK, Zarate P. Closing the gaps: the impact of inpatient detoxification and continuity of care on client outcomes. Journal Psychoactive Drugs. 2010;42(sup6):303-14.

12. Lee MT, Horgan CM, Garnick DW, Acevedo A, Panas L, Ritter GA, et al. A performance measure for continuity of care after detoxification: Relationship with outcomes. J Subst Abuse Treat. 2014;47(2):130-9.

13. Cumming C, Troeung L, Young JT, Kelty E, Preen DB. Barriers to accessing methamphetamine treatment: A systematic review and meta-analysis. Drug Alcohol Depend. 2016;168:263-73.

14. Brecht ML, Lovinger K, Herbeck DM, Urada D. Patterns of treatment utilization and methamphetamine use during first 10 years after methamphetamine initiation. J Subst Abuse Treat. 2013;44(5):548-56.

15. Ezard N, Dunlop A, Hall M, Ali R, McKetin R, Bruno R, et al. LiMA: A study protocol for a randomised, double-blind, placebo controlled trial of lisdexamfetamine for the treatment of methamphetamine dependence. BMJ Open. 2018;8(7).

16. Ezard N, Dunlop A, Clifford B, Bruno R, Siefried K, Carr A, et al. Safety of oral lisdexamfetamine in adults with methamphetamine dependence: a dose-escalating phase-2 study. The College on Problems of Drug Dependence 81st Annual Scientific Meeting; San Antonio, US2019.

17. Siefried KJ, Acheson LS, Lintzeris N, Ezard N. Pharmacological Treatment of Methamphetamine/Amphetamine Dependence: A Systematic Review. CNS Drugs. 2020.

18. Brecht M-L, von Mayrhauser C, Anglin MD. Predictors of relapse after treatment for methamphetamine use. Journal Psychoactive Drugs. 2000;32(2):211-20.

19. Wodarz N, Krampe-Scheidler A, Christ M, Fleischmann H, Looser W, Schoett K, et al. Evidence-Based Guidelines for the Pharmacological Management of Acute Methamphetamine-Related Disorders and Toxicity. Pharmacopsychiatry. 2017;50(03):87-95.

20. Pennay AE, Lee NK. Putting the call out for more research: the poor evidence base for treating methamphetamine withdrawal. Drug Alcohol Rev. 2011;30(2):216-22.

21. Srisurapanont M, Jarusuraisin N, Jittiwutikan J. Amphetamine withdrawal: I. Reliability, validity and factor structure of a measure. ANZJP. 1999;33(1):89-93.

22. Lee JW, Brown ES, Perantie DC, Bobadilla L. A comparison of single-item visual analog scales with a multiitem likert-type scale for assessment of cocaine craving in persons with bipolar disorder. Addict Disord Their Treat. 2002;1(4):140-2.

23. Lee NK, Rawson RA. A systematic review of cognitive and behavioural therapies for methamphetamine dependence. Drug Alcohol Rev. 2008;27(3):309-17.

24. Montagna P, Gambetti P, Cortelli P, Lugaresi E. Familial and sporadic fatal insomnia. Lancet Neurol. 2003;2(3):167-76.

25. Malow BA. Sleep deprivation and epilepsy. Epilepsy Curr. 2004;4(5):193-5.

26. Stickgold R. A memory boost while you sleep. Nature. 2006;444(7119):559-60.

27. Lipinska G, Timol R, Thomas KGF. The implications of sleep disruption for cognitive and affective processing in methamphetamine abuse. Med Hypotheses. 2015;85(6):914-21.

28. Grant KM, LeVan TD, Wells SM, Li M, Stoltenberg SF, Gendelman HE, et al. Methamphetamine-Associated Psychosis. J Neuroimmune Pharmacol. 2012;7(1):113-39.

29. Perez AY, Kirkpatrick MG, Gunderson EW, Marrone G, Silver R, Foltin RW, et al. Residual effects of intranasal methamphetamine on sleep, mood, and performance. Drug Alcohol Depend. 2008;94(1):258-62.

30. Mahoney JJ, De La Garza R, Jackson BJ, Verrico CD, Ho A, Iqbal T, et al. The relationship between sleep and drug use characteristics in participants with cocaine or methamphetamine use disorders. Psychiatry Res. 2014;219(2):367-71.

31. Meredith CW, Jaffe C, Ang-Lee K, Saxon AJ. Implications of chronic methamphetamine use: a literature review. Harvard Rev Psychiatry. 2005;13(3):141-54.

32. Cruickshank CC, Montebello ME, Dyer KR, Quigley A, Blaszczyk J, Tomkins S, et al. A placebo-controlled trial of mirtazapine for the management of methamphetamine withdrawal. Drug Alcohol Rev. 2008;27(3):326-33.

33. Bailey RC, Jackson D. A pharmacological study of changes in central nervous system receptor responsiveness after long-term dexamphetamine and apomorphine administration. Psychopharmacology. 1978;56(3):317-26.

34. Pennick M. Absorption of lisdexamfetamine dimesylate and its enzymatic conversion to d-amphetamine. Neuropsychiatr Dis Treat. 2010;6:317.

35. Krishnan SM, Pennick M, Stark JG. Metabolism, distribution and elimination of lisdexamfetamine dimesylate. Clin Drug Investig. 2008;28(12):745-55.

36. Krishnan SM, Stark JG. Multiple daily-dose pharmacokinetics of lisdexamfetamine dimesylate in healthy adult volunteers. CMRO. 2008;24(1):33-40.

37. Jasinski D, Krishnan S. Abuse liability and safety of oral lisdexamfetamine dimesylate in individuals with a history of stimulant abuse. J Psychopharmacol. 2009;23(4):419-27.

38. Ashok AH, Mizuno Y, Volkow ND, Howes OD. Association of stimulant use with dopaminergic alterations in users of cocaine, amphetamine, or methamphetamine: a systematic review and meta-analysis. JAMA Psychiatry. 2017;74(5):511-9.

39. Tata DA, Yamamoto BK. Interactions between methamphetamine and environmental stress: role of oxidative stress, glutamate and mitochondrial dysfunction. Addiction. 2007;102:49-60.

40. Chou Y-H, Huang W-S, Su T-P, Lu R-B, Wan F-J, Fu Y-K. Dopamine transporters and cognitive function in methamphetamine abuser after a short abstinence: A SPECT study. Eur Neuropsychopharmacol. 2007;17(1):46-52.

41. McCann UD, Kuwabara H, Kumar A, Palermo M, Abbey R, Brasic J, et al. Persistent cognitive and dopamine transporter deficits in abstinent methamphetamine users. Synapse. 2008;62(2):91-100.

42. Posner K, Brown GK, Stanley B, Brent DA, Yershova KV, Oquendo MA, et al. The Columbia–Suicide Severity Rating Scale: initial validity and internal consistency findings from three multisite studies with adolescents and adults. AmJ Psychiatry. 2011;168(12):1266-77.

43. Ward MF. The Wender Utah Rating Scale: an aid in the retrospective diagnosis of childhood attention deficit hyperactivity disorder. Am J Psychiatry. 1993;150:885-.

44. Gossop M, Darke S, Griffiths P, Hando J, Powis B, Hall W, et al. The Severity of Dependence Scale (SDS): psychometric properties of the SDS in English and Australian samples of heroin, cocaine and amphetamine users. Addiction. 1995;90(5):607-14.

45. McKetin R, Lubman DI, Baker AL, Dawe S, Ali RL. Dose-related psychotic symptoms in chronic methamphetamine users: evidence from a prospective longitudinal study. JAMA Psychiatry. 2013;70(3):319-24.

46. Overall JE, Gorham DR. The brief psychiatric rating scale. Psychol Rep. 1962;10(3):799-812.

47. Yiengprugsawan V, Seubsman S-a, Khamman S, Lim L-Y, Sleigh AC, Team TCS. Personal wellbeing index in a national cohort of 87,134 Thai adults. Soc Indic Res. 2010;98(2):201-15.

48. Mitchell PH, Powell L, Blumenthal J, Norten J, Ironson G, Pitula CR, et al. A short social support measure for patients recovering from myocardial infarction: the ENRICHD Social Support Inventory. J Cardiopulm RehabilPrev. 2003;23(6):398-403.

49. Robinson SM, Sobell LC, Sobell MB, Leo GI. Reliability of the Timeline Followback for cocaine, cannabis, and cigarette use. Psychol Addict Behav. 2014;28(1):154.

50. Atkinson MJ, Sinha A, Hass SL, Colman SS, Kumar RN, Brod M, et al. Validation of a general measure of treatment satisfaction, the treatment satisfaction questionnaire for medication (TSQM), using a national panel study of chronic disease. Health Qual Life Outcomes. 2004;2.

51. Carney CE, Buysse DJ, Ancoli-Israel S, Edinger JD, Krystal AD, Lichstein KL, et al. The Consensus Sleep Diary: Standardizing Prospective Sleep Self-Monitoring. Sleep. 2012;35(2):287-302.

52. Shire Australia Pty Ltd. Vyvanse (lisdexamfetamine) [prouduct information] 2013 [updated May 2020. Available from: https://www.ebs.tga.gov.au/ebs/picmi/picmirepository.nsf/pdf?OpenAgent&id=CP-2013-PI-02051-1&d=202007301016933.

53. Biel JH, Bopp BA. Amphetamines: Structure-Activity Relationships. In: Iversen LL, Iversen SD, Snyder SH, editors. Stimulants. Boston, MA: Springer US; 1978. p. 1-39.

54. Martin P, Dirks B, Gertsik L, Walling D, Stevenson A, Corcoran M, et al. Safety and pharmacokinetics of lisdexamfetamine dimesylate in adults with clinically stable schizophrenia a randomized, double-blind, placebo-controlled trial of ascending multiple doses. J Clin Psychopharmacol. 2014;34(6):682-9.

55. National Institute of Allergy and Infectious Diseases, Division of AIDS. Division of AIDS (DAIDS) Table for Grading the Severity of Adult and Pediatric Adverse Events, Corrected Version 2.1. In: US Department of Health and Human Services, editor. 2018.

56. Council for International Organizations of Medical Sciences. Management of safety information from clinical trials: report of CIOMS Working Group VI. 2005.

57. Hertzog MA. Considerations in determining sample size for pilot studies. Res Nursing Health. 2008;31(2):180-91.

58. Julious SA. Sample size of 12 per group rule of thumb for a pilot study. Pharm Stat. 2005;4(4):287-91.

59. The National Health and Medical Research Council, The Australian Research Council, Universities Australia. National Statement on Ethical Conduct in Human Research. Commonwealth of Australia, Canberra2007 (updated 2018).
